# Giant Oncocytic Sinonasal Papilloma: A Rare Case

**DOI:** 10.3390/diagnostics16162632

**Published:** 2026-08-19

**Authors:** André Gustavo da Silva Corrêa, Adriane Xavier de Morais, Lucio Murilo dos Santos, Carlos Castilho Borges, Ana Lia Anbinder, Renata Falchete do Prado

**Affiliations:** 1 Department of Biosciences and Oral Diagnosis, Institute of Science and Technology, São Paulo State University (UNESP), Av. Eng. Francisco José Longo, 777, Jardim São Dimas, São José dos Campos 12245-000, SP, Brazil; andre.g.correa@unesp.br (A.G.d.S.C.); adriane.xavier@unesp.br (A.X.d.M.); ana.anbinder@unesp.br (A.L.A.); 2 Department of Diagnosis and Surgery, Institute of Science and Technology, São Paulo State University (UNESP), São José dos Campos 12245-000, SP, Brazil; lucio.murilo@unesp.br; 3 Private Practice, São José dos Campos 12245-280, SP, Brazil; dr.carloscastilho@gmail.com

**Keywords:** paranasal sinus neoplasms, papilloma, oncocytic cells

## Abstract

Sinonasal papilloma is a rare benign neoplasm arising from the Schneiderian epithelium lining the nasal cavity and paranasal sinuses. It is classified into three histological subtypes: exophytic, inverted, and oncocytic, the latter being the least common. We report a case of a giant oncocytic sinonasal papilloma treated by endoscopic surgery, highlighting the diagnostic approach and comparing the findings with the existing literature. Histopathological examination confirmed the diagnosis. Immunohistochemical analysis demonstrated p53 positivity in both the cytoplasm and nucleus, and Ki-67 showed a low labeling index. The patient underwent complete excision via an endoscopic approach. Notably, the lesion was larger than those typically described in the literature. An 18-month follow-up revealed no complications or evidence of recurrence. It is important to bear in mind that a combined tomographic and histopathological evaluation is essential for accurate diagnosis and therapeutic approach.

**Figure 1:**
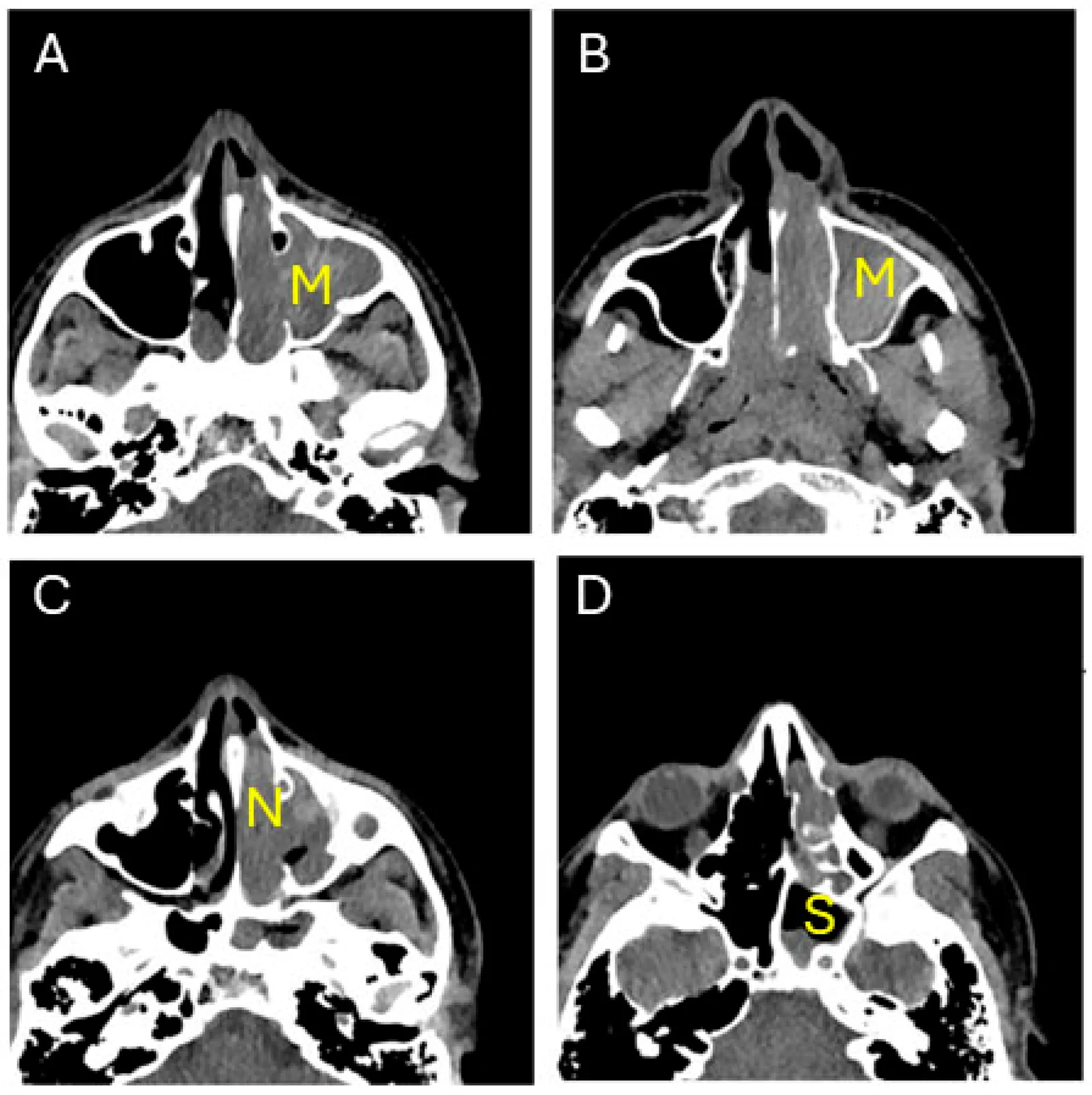
A 44-year-old male presented with a six-month history of nasal obstruction, without associated pain. Written informed consent was obtained from the patient for participation in this study and for the publication of this case report and any accompanying images. He denied smoking and alcohol consumption. The diagnostic workup followed a sequential approach: initial clinical examination was followed by computed tomography (CT), incisional biopsy with histopathological diagnosis, referral for definitive treatment, surgical excision, and postoperative follow-up. CT in axial plane revealed a soft tissue density mass occupying almost the entire left maxillary sinus (M) (**A**,**B**), extending into the left nasal cavity (N) (**C**) and nasopharynx. The lesion obliterated the left ostiomeatal complex and frontoethmoidal recess and was associated with thinning of the medial and inferior walls of the left orbit. Mucosal thickening and/or retained secretions were also observed in the right maxillary sinus, left ethmoid air cells, left frontal sinus, and left sphenoid sinus (S) (**D**). No extension into the pterygopalatine fossae was identified. Bone remodeling, enlargement of the natural ostium of the maxillary sinus, and focal changes suggestive of osteitis were noted (characterized by cortical thickening and sclerosis of the sinus walls, consistent with a long-standing underlying process). The absence of extensive bone destruction on imaging suggested a benign lesion (differential diagnosis of antrochoanal polyp or Killian’s polyp); however, definitive diagnosis relied on histopathological examination. An incisional biopsy was performed, with a diagnosis of oncocytic sinonasal papilloma. Although magnetic resonance imaging (MRI) is considered the most accurate imaging modality for evaluating the soft-tissue extent of sinonasal tumors [1], only CT imaging was available in the present case. CT nevertheless provided adequate information regarding lesion extent, bony remodeling, and preoperative surgical planning, which were sufficient for the clinical management of this patient. The absence of MRI is acknowledged as a limitation of the present report. The patient was referred to an otolaryngologist, who performed tumor excision using an endoscopic approach. Sinonasal papillomas are rare benign neoplasms of Schneiderian epithelial origin affecting the nasal cavity and paranasal sinuses. This lesion was first described in 1854 by Ward [2]. Since then, various terms have been used to refer to the three histological subtypes, which were officially recognized by the World Health Organization (WHO) in 2005 and remain in use today (last edition published in 2022) [3]. The distinct histological subtypes include the inverted and oncocytic variants, which primarily affect the paranasal cavity and maxillary sinus, and the exophytic variant, which has a predilection for the nasal septum [4,5]. The etiology of these lesions remains unclear; however, there is evidence suggesting the involvement of Human papillomavirus (HPV) infection and tobacco use in their pathogenesis [3]. The rarest subtype is the oncocytic variant, with an incidence ranging from 3% to 5%, followed by the exophytic variant, and lastly the inverted variant, which is the most common [5]. Most published cases of inverted sinonasal papillomas preferentially affect men in their fifth decade of life and are typically unilateral, whereas oncocytic sinonasal papillomas do not appear to have a gender predilection and tend to occur in older individuals [5,6]. The causes and risk factors associated with the development of oncocytic sinonasal papillomas remain unconfirmed. Notably, HPV infection is not considered an etiological factor [7]. Among the three subtypes of sinonasal papilloma, the oncocytic and inverted types exhibit a higher likelihood of malignant transformation. However, the factors that modulate this risk remain undefined. A 2020 study that analyzed 137 cases of sinonasal papilloma, examining recurrence and potential for malignant transformation, demonstrated that both recurrence and transformation were more frequent in oncocytic sinonasal papillomas [4]. The malignancy most commonly associated with this subtype was squamous cell carcinoma [8]. Complete excision provides a substantial advantage in reducing recurrence rates; therefore, every effort should be directed toward complete disease excision during the initial procedure [9]. Oncocytic sinonasal papillomas present clinically as unilateral tumors that may remain confined within the nasal cavity or may involve the osteomeatal complex, ethmoid sinus, maxillary sinus, sphenoid sinus, and frontal sinus, with or without concomitant involvement of the nasal cavity [5]. The present report describes an extensive sinonasal papilloma occupying almost the entire left maxillary sinus, extending into the left nasal cavity and nasopharynx. The location is in agreement with a series of 33 cases of oncocytic sinonasal papillomas, which also demonstrated a higher incidence in the maxillary sinus (78.8%) [8]. In the literature, most reported cases range from 10 to 20 cm^3^ in volume [6,10]. The present lesion is larger than most reported cases. The main complaints among patients with oncocytic sinonasal papilloma are unilateral nasal obstruction and intermittent epistaxis. Typically, the condition is painless, and symptoms may persist for years [11]. In the present case, the patient sought medical attention after six months of nasal obstruction without other complaints. Physical examination and imaging typically yield differential diagnoses such as nasal polyps and malignant tumors; however, they are not useful for distinguishing between histological subtypes of sinonasal papillomas. Imaging primarily serves to determine the tumor’s extent and precise localization, since it cannot differentiate papillomas from other lesions in the same region, such as chronic sinusitis [5]. In this report, the CT mimicked Killian’s polyp, with a mass filling the almost the entire left maxillary sinus, extending into the left nasal cavity and nasopharynx. Contrary to the findings previously reported by Shafqat et al. [12], the underlying bone exhibited signs of osteitis in the present case. Diagnostic imaging can aid in the identification of inverted papilloma when a polypoid sinonasal mass originates from the lateral nasal wall. CT typically will reveal a nonspecific soft-tissue mass centered in the middle meatus, often accompanied by bone remodeling [13] or bone erosions, which may be associated with malignancy. Nonetheless, such associations must be confirmed through histopathological analysis [14]. In this case, the CT were suggestive of a benign lesion, evidenced by the absence of bone destruction, which was subsequently confirmed through incisional biopsy—an essential step in surgical planning. In the present case, after fixation, routine histological processing was conducted and specimen was paraffin embedding. For morphological analysis, 5 μm sections were stained with hematoxylin and eosin (HE).

**Figure 2:**
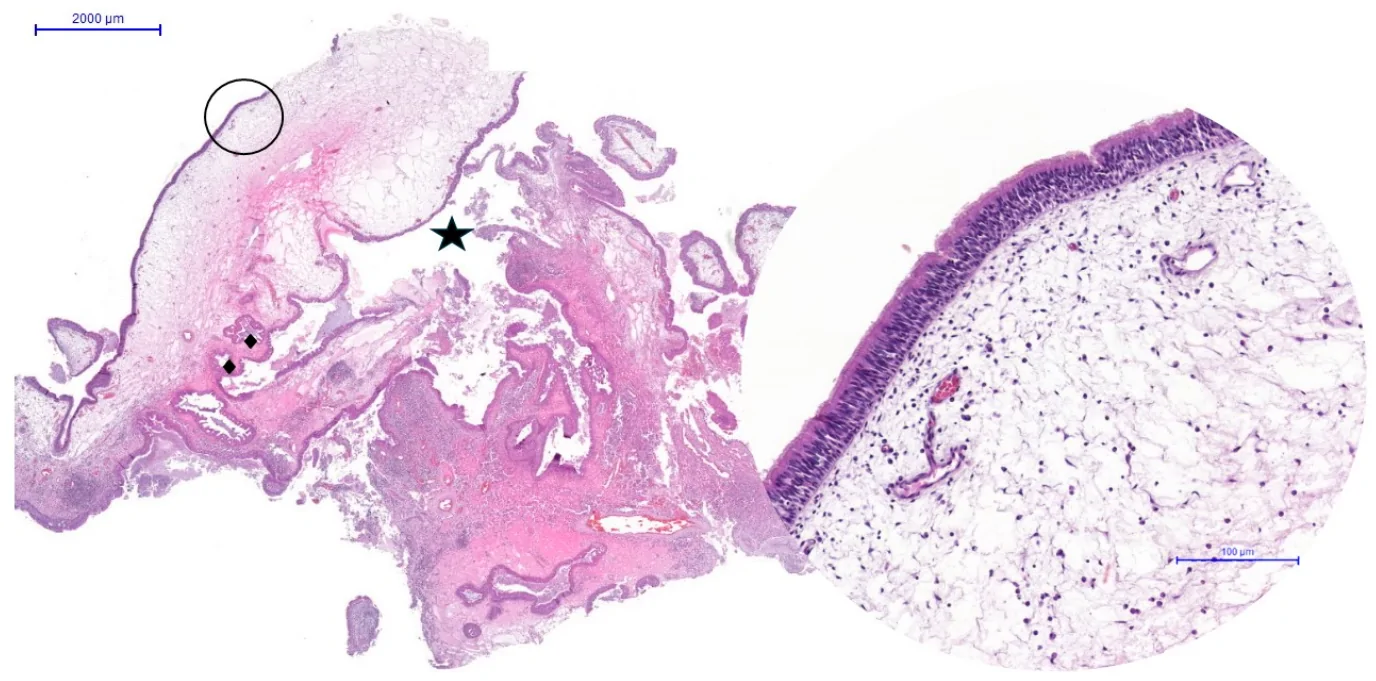
Histopathological features of oncocytic sinonasal papilloma. The microscopic examination confirmed the presence of respiratory epithelium at the lesion site, involving the maxillary sinus and nasal cavity. Histological sections revealed fragments of nasal sinus mucosa exhibiting a benign epithelial neoplasm composed of macro (★) and microcystic areas (⬥). There was no evidence of nuclear or cellular atypia, including abnormal mitotic figures, and the basement membrane was preserved as previously described by Nishimura et al. [15].

**Figure 3:**
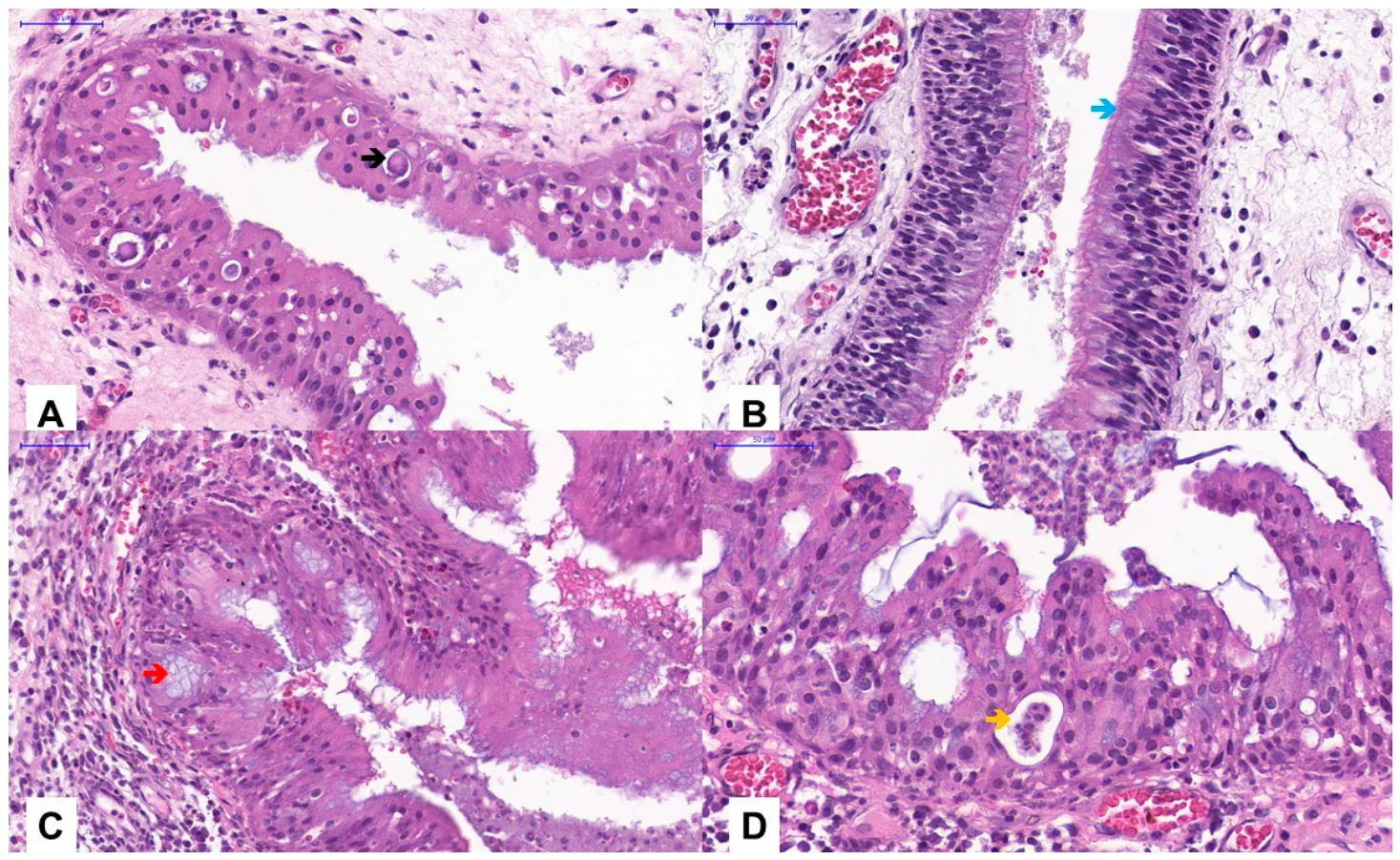
Detailed epithelial features. The epithelium showed extensive oncocytic lining (**A**) and areas of mucin microcysts ((**A**) black arrow), pseudostratified columnar epithelium ((**B**) blue arrow), mucous cells ((**C**) red arrow), and ciliated cells in various stages of regression (3B and 3C). Microabscesses were occasionally observed within the epithelium ((**D**) yellow arrow). Afzalzadeh et al. [16] presented in one high magnification image oncocytic cylindrical cells with abundant eosinophilic cytoplasm, intraepithelial mucin filled cysts with neutrophilic microabscess when describing a middle ear oncocytic papilloma.

**Figure 4:**
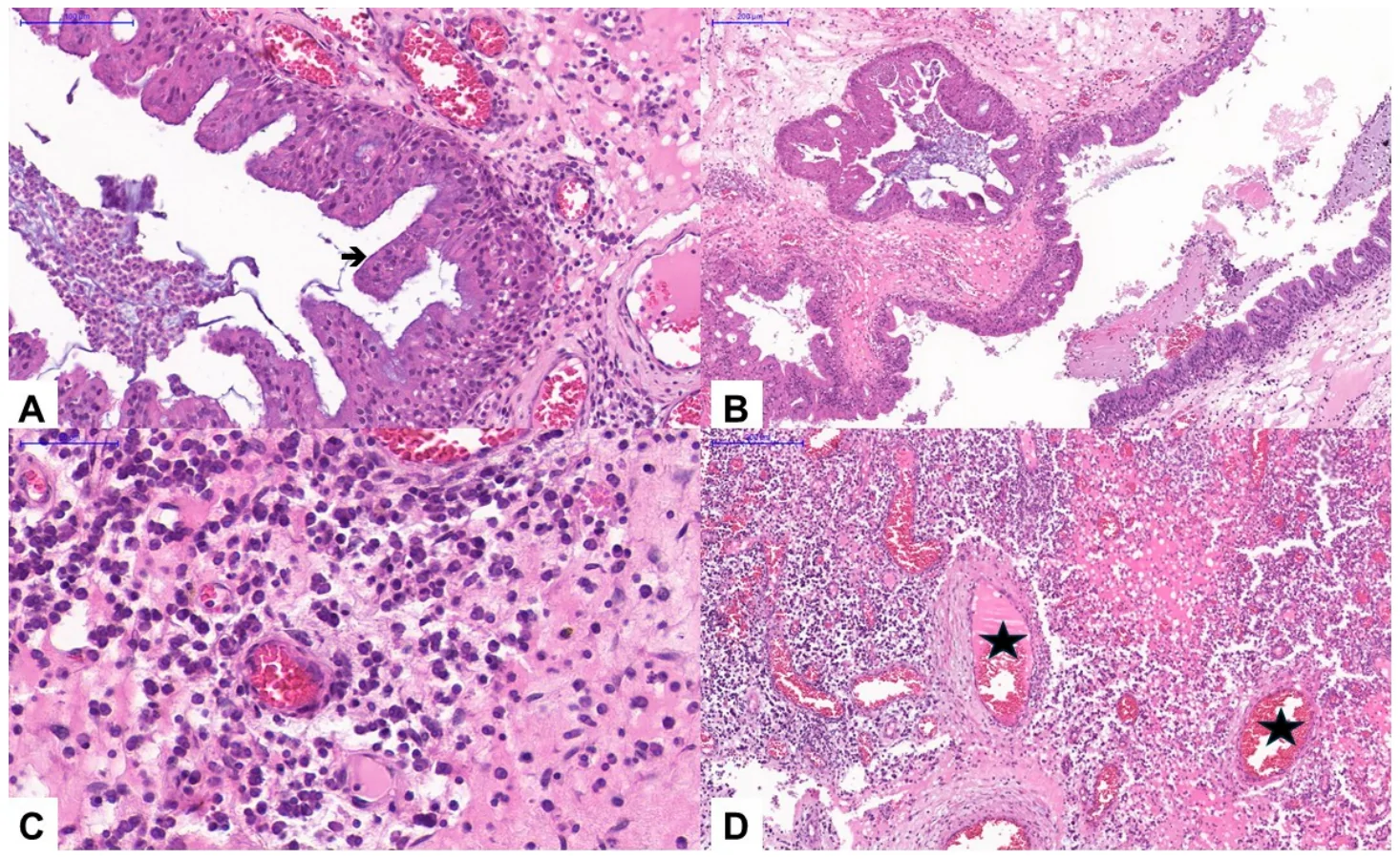
Tumor proliferation with associated stromal inflammatory response. Oncocytic lining epithelium presented both papillary exophytic ((**A**) black arrow) and endophytic growth. The underlying connective tissue showed intense edema (**B**) and a mononuclear inflammatory infiltrate predominantly composed of plasma cells (**C**). Blood vessels were dilated and congested ((**D**) black star). The cystic spaces contained mucopurulent material with mononuclear and polymorphonuclear inflammatory cells (**B**). A noteworthy microscopic feature in the present case was the presence of cysts containing mucous material. Although MRI was not performed in this patient, previous studies have reported that oncocytic papillomas are more likely than inverted papillomas to exhibit abundant mucus and multiple cystic changes on MRI (94.1% vs. 17.6%) [4]. The three subtypes of sinonasal papillomas (exophytic, inverted, and oncocytic) appear clinically similar but differ histologically. The case reported here showed a high proportion of epithelium with papillary projections and oncocytic characteristics, along with intraepithelial microabscesses and mucinous microcysts (classic features described in the literature) [7]. One of the main objectives of histopathological examination is to exclude the presence of associated carcinoma, given that these lesions have malignant transformation potential. In 10% of inverted papillomas, some degree of dysplasia is observed, which does not necessarily indicate carcinoma but warrants a more thorough evaluation, as approximately 9% of inverted papillomas are associated with carcinoma [11]. The most common malignancy associated with papillomas is squamous cell carcinoma. In the study by Maisch et al. [4], the rate of malignant transformation in oncocytic sinonasal papillomas was 33.3%. The p53 gene is a tumor suppressor involved in DNA repair, cell cycle arrest, cellular senescence, apoptosis, cellular differentiation, and metabolism. One of its key functions is to halt the progression of cells with DNA damage during the G1 phase of the cell cycle [17]. Mutations in the TP53 (gene located at chromosome 17p13.1) and CDKN2A (cyclin-dependent kinase inhibitor 2A) genes are associated with papillomas that have undergone malignant transformation [18]. We performed immunohistochemical analysis on 3 μm sections placed on slides previously treated with 3-aminopropyltriethoxysilane. Deparaffinization was done with two xylene changes, the first for 30 min at 60 °C and the second at room temperature for 20 min. Sections were then hydrated in decreasing concentrations of ethanol (100%, 90%, 70%, and 50%) and washed in running water. Formalin pigment removal was performed by immersion in water and ammonium hydroxide solution. Antigen retrieval was performed using a 10 mM citric acid buffer (pH 6.0) in a water bath for p53, and an Ethylenediaminetetraacetic acid (EDTA) buffer in a pressure cooker at 124 °C for 1 min for Ki-67. Slides were washed with running and distilled water. Endogenous peroxidase activity was blocked with H_2_O_2_ and methanol (1:1). Sections were washed in running water and incubated twice in Tris buffer (pH 7.4) for five minutes each. Nonspecific antibody blocking was done by incubation in 1% bovine serum albumin (BSA) solution (Sigma-Aldrich, Darmstadt, Germany) for one hour. Slides were then incubated for 1 h with anti-p53 primary antibody (DO7, Dako S/A, Glostrup, Denmark) (1:25) and anti-Ki-67 primary antibody (SP6, SpringBioscience, Pleasanton, CA, USA) (1:25). After primary antibody incubation, sections were washed in Tris buffer and incubated for 30 min at 37 °C with the Envision detection system (Agilent Technologies, Santa Clara, CA, USA). Slides were washed and incubated with diaminobenzidine (DAB + Substrate Chromogen System Dako Denmark S/A) for 3 min. Slides were then washed, counterstained with Mayer’s hematoxylin, dehydrated in ethanol, cleared in xylene, and mounted with Permount.

**Figure 5:**
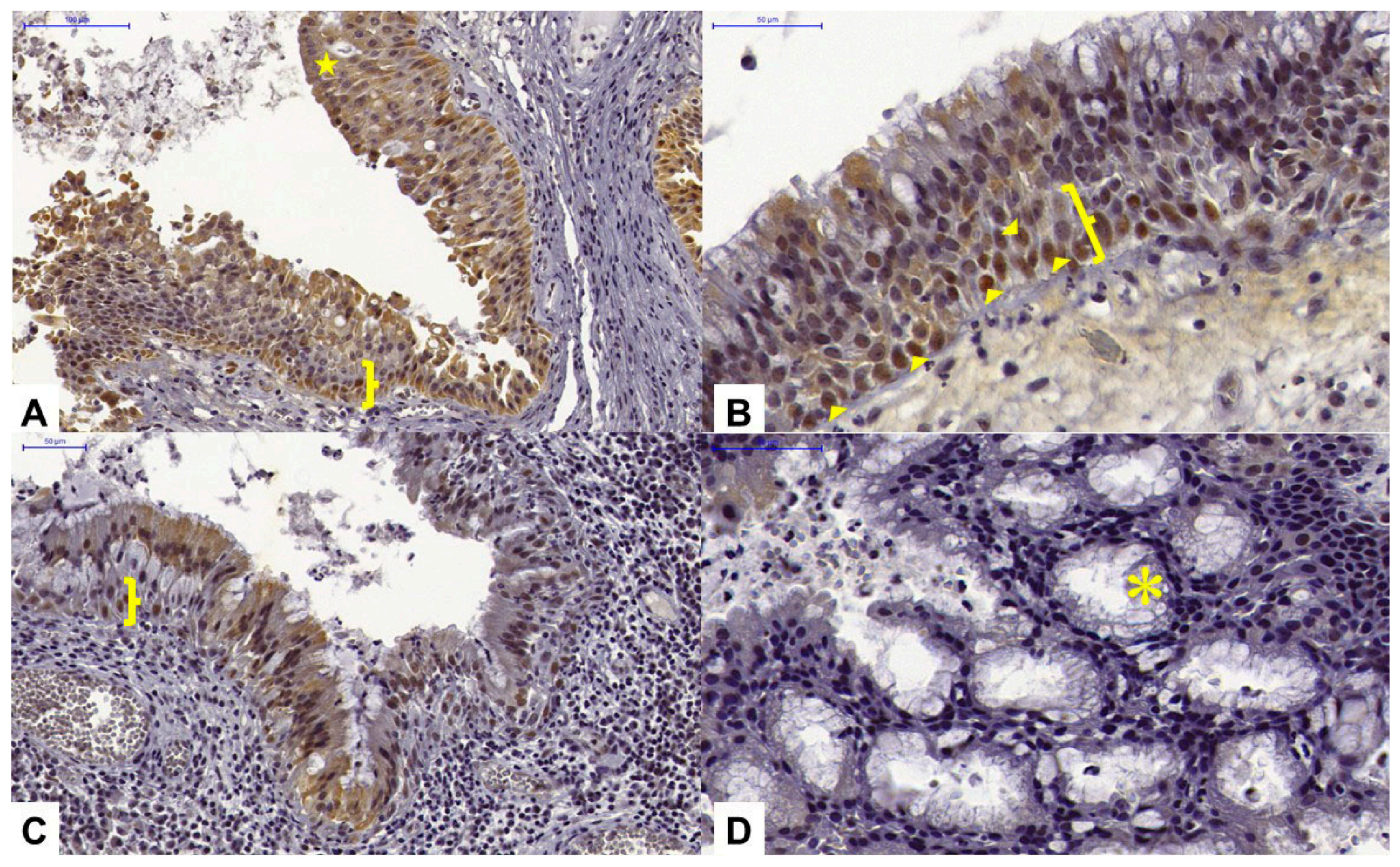
(**A**,**B**) Patchy nuclear immunoreactivity (yellow arrowhead) predominantly confined to the basal and parabasal epithelial layers (in braces), consistent with the physiological wild-type p53 expression pattern. No diffuse strong nuclear overexpression or complete absence of nuclear staining (null pattern), both recognized surrogate immunohistochemical patterns associated with TP53 mutation [18], was observed (**A**–**C**), mucous cells were negative (* (**D**) scale bar = 50 µm). Diffuse cytoplasmic staining is also observed in oncocytic epithelial cells (yellow star). Because p53 is physiologically a nuclear protein, this cytoplasmic immunoreactivity should be interpreted with caution and most likely reflects the abundant mitochondria-rich cytoplasm characteristic of oncocytes, a recognized source of artifactual staining with anti-p53 antibodies, rather than abnormal p53 protein accumulation or TP53 mutation. Therefore, the observed cytoplasmic staining should not be interpreted as a specific molecular feature without complementary molecular validation. (**D**) Mucous and inflammatory cells are negative for p53. Importantly, epithelial cells exhibiting either nuclear or cytoplasmic p53 immunoreactivity showed no cytological atypia or architectural abnormalities indicative of malignant transformation. Consistent with these findings, Finkelstein et al. [19] reported nuclear p53 immunoreactivity in benign sinonasal papillomas, whereas diffuse nuclear overexpression was more frequently associated with malignant transformation, supporting the concept that the immunohistochemical expression pattern, rather than the mere presence of nuclear staining, is more closely associated with malignant progression. The present case demonstrates a wild-type staining pattern characterized by heterogeneous, predominantly basal/parabasal nuclear labeling without diffuse overexpression or null staining, supporting the benign nature of the lesion [18]. The literature includes assessments of EGFR (epidermal growth factor receptor) and KRAS (Kirsten rat sarcoma viral oncogene) gene mutations. EGFR mutations have been identified in all cases of inverted papilloma associated with carcinoma, whereas KRAS mutations were found in oncocytic papillomas associated with carcinoma [20]. EGFR mutations were present in 78% of inverted papilloma cases. No KRAS mutations were detected in these cases [20]. Conversely, KRAS mutations were identified in 82% of oncocytic papilloma cases, while EGFR mutations were absent [4]. A limitation of the present report is the lack of molecular characterization. Although oncocytic sinonasal papillomas frequently harbor KRAS exon 2 mutations [20], molecular testing was not available at our institution and is not routinely performed in many public healthcare and academic centers in Latin America. Furthermore, the diagnosis was supported by the characteristic clinicopathological features of the lesion, and molecular findings would not have altered patient management. Nevertheless, future studies incorporating molecular analyses may further contribute to the understanding and classification of these uncommon tumors.

**Figure 6:**
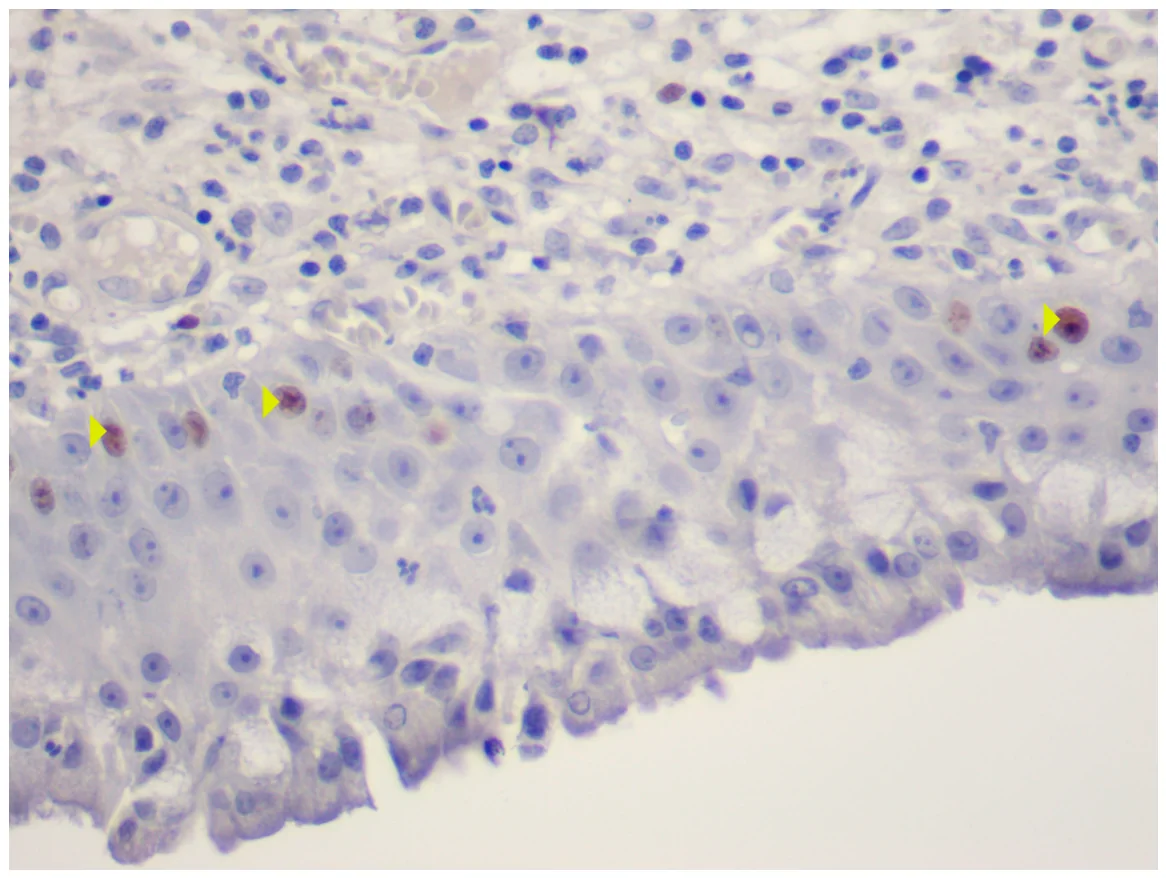
Representative photomicrograph of the epithelial area exhibiting the highest density of Ki-67-positive nuclei (arrowheads—hot spot). Nuclear immunoreactivity is predominantly observed in basal and parabasal epithelial cells, with occasional positive nuclei extending into the lower suprabasal layers. Most epithelial cells are negative for Ki-67, indicating preservation of the typical basal/parabasal proliferative compartment. Hematoxylin counterstain. In tumour, 500 neoplastic cells were counted at randomly chosen fields at 400× magnification. The growth fraction of the tumor was expressed as the percentage of positively stained neoplastic nuclei in the total number of nuclei counted according Katori et al. [21]. The Ki-67 labeling index was 5.6% (28/500 cells), which is lower than the mean values reported by Katori et al. for inverted sinonasal papillomas with mild (8.5%) and moderate (9.0%) dysplasia, supporting a low proliferative activity. Despite emerging insights into the molecular mechanisms underlying oncogenesis in sinonasal papillomas, the pathways involved in malignant transformation into sinonasal carcinoma remain relatively poorly understood [22]. The standard treatment for all types of sinonasal papillomas is complete surgical excision. The choice of surgical technique depends on imaging findings that define lesion extent and characteristics. Complete resection must include affected mucosa and mucoperiosteum to minimize recurrence risk. Closer follow-up is recommended given its higher risk of malignant transformation compared to other subtypes [8]. The patient of the present report has been under follow-up for one and a half years, without complications or lesion recurrence. Although no recurrence has been observed during the current follow-up period, the patient remains under clinical surveillance. In extensive sinonasal papillomatous lesions, postoperative outcome depends not only on the histopathological diagnosis but also on accurate delineation of the lesion extent and complete removal of the involved mucosa. Therefore, careful long-term follow-up is recommended to allow early detection of recurrence [23,24]. Various surgical approaches have been employed; nevertheless, the endoscopic technique is currently the standard of care as it reduces surgical morbidity and significantly shortens operative time [5]. Postoperative radiotherapy is indicated when papillomas are associated with squamous cell carcinoma or when complete surgical excision is not feasible [4]. CT evaluation is essential for determining lesion extent and guiding surgical planning, whereas histopathological analysis is required for definitive diagnosis and assessment of malignant transformation. Careful long-term follow-up is recommended because of the risk of recurrence.

## Data Availability

The data supporting the findings of this study are available from the corresponding author upon request.
